# HDL attenuates Ang II–AT1R–EGFR signaling and reverses vascular remodeling in spontaneously hypertensive rats

**DOI:** 10.3389/fphar.2025.1617420

**Published:** 2025-07-29

**Authors:** Aishah Al-Jarallah, Samah Kalakh, Saghir Akhtar, Mariam H. M. Yousif

**Affiliations:** ^1^ Department of Biochemistry, College of Medicine, Kuwait University, Kuwait City, Kuwait; ^2^ College of Medicine, QU Health, Qatar University, Doha, Qatar; ^3^ Department of Pharmacology and Toxicology, College of Medicine, Kuwait University, Kuwait City, Kuwait

**Keywords:** HDL, angiotensin II, SR-BI, vascular remodeling, proliferation

## Abstract

**Background:**

Angiotensin II (Ang II) signaling via angiotensin II type 1 receptor (AT1R) and transactivation of epidermal growth factor receptor (EGFR) enhances vascular smooth muscle cell (VSMC) proliferation and contributes to vascular remodeling evident in spontaneously hypertensive rats (SHR) aorta. Although high-density lipoprotein (HDL) has been shown to lower blood pressure in SHR, the underlying mechanism(s) remain incompletely understood. We propose that HDL attenuates Ang II–AT1R–EGFR signaling and reverses vascular remodeling in SHR.

**Methods:**

Wistar Kyoto rats (WKY) and SHR were treated with HDL for 1 week. Vascular remodeling was histologically examined. VSMC proliferation and the expression levels of AT1R, EGFR, extracellular signal regulated kinases 1/2 (ERK1/2), scavenger receptor class B type-I (SR-BI) and its adaptor protein PDZK1 were examined by immunofluorescence. VSMC proliferation was further examined *in vitro*.

**Results:**

HDL treatment reduced blood pressure, increased the production of nitric oxide, increased aortic lumen diameter, reduced media thickness to lumen diameter ratio, decreased collagen contents in SHR. Furthermore, HDL treatment decreased the number of proliferating VSMCs and α-smooth muscle actin, reduced the expression of AT1R and EGFR and increased the expression of SR-BI adaptor protein, PDZK1, in SHR aortas. In isolated VSMCs, HDL attenuated Ang II-induced proliferation by reducing AT1R expression and decreasing Ang II-induced transactivation of EGFR. HDL effects were SR-BI dependent and were mimicked by different HDL subpopulations, reconstituted HDL, and lipid free apolipoprotein A-I.

**Conclusion:**

HDL attenuates Ang II–AT1R–EGFR signaling, reduces VSMC proliferation, and reverses vascular remodeling in SHR. HDL modulation of vascular remodeling could be one mechanism by which HDL reduces blood pressure in SHR.

## Graphical Abstract

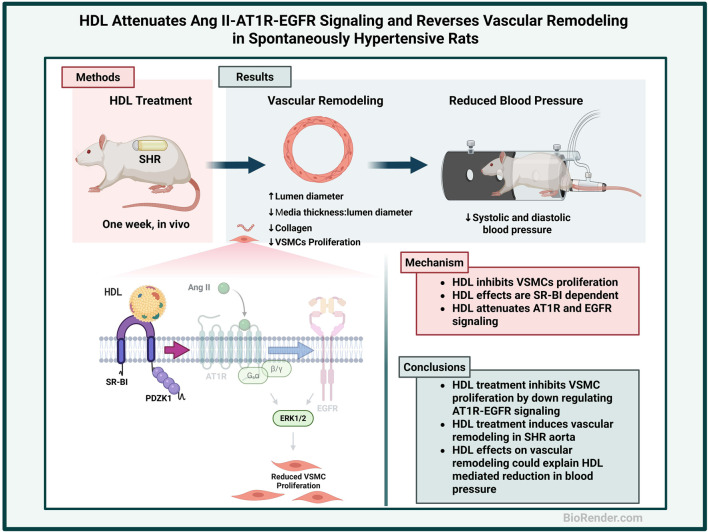

## Introduction

Hypertension is a major global health concern that affects more than one billion adults worldwide (Carey et al., 2022) and continues to be the most prevalent risk factor for cardiovascular diseases (GBDRFC, 2018). Antihypertensive therapies demonstrated clinical benefit in individuals at high cardiovascular risk, as well as in patients with established cardiovascular disease, irrespective of their baseline blood pressure status. In addition, an inverse relationship exists between high-density lipoprotein (HDL) cholesterol levels and the risk of coronary heart disease (CHD) (Assmann et al., 1996; Barr et al., 1951; Boyd and Oliver, 1957; Brown and Goldstein, 1983; Gordon and Rifkind, 1989). Reduced serum HDL cholesterol (HDL-C) and hypertension are indicators of metabolic syndrome and important predictors of acute myocardial infarction (Oyama et al., 2006). Increased blood pressure eliminated the protective effects of HDL against CHD and stroke (Hamad et al., 2001). Hypertension and anti-hypertensive medications altered HDL function and composition (Ozerova et al., 2007). Patients with arterial hypertension had reduced contents of HDL phospholipids and altered phospholipid composition. However, the association between HDL-C levels and hypertension has been inconsistently reported in the literature (Kawamoto et al., 2012; Ozerova et al., 2007; Wu et al., 2022). HDL-C levels have been inversely associated with the risk of hypertension (Kunutsor et al., 2017; Hwang et al., 2019) and a combination of high-normal blood pressure with low HDL-C has been linked to increased mortality (Kim et al., 2011). Nonetheless, increased HDL-C enhanced cardiovascular risk in hypertensive male, but not female, patients (Trimarco et al., 2022), suggesting a complex role of HDL in hypertension. This underscores the importance of HDL function and composition, over absolute HDL-C levels, in determining cardiovascular risk.

Vascular remodeling, characterized by enhanced collagen deposition, increased intima/media thickness and alterations in elastin/collagen ratio, is a well-documented feature of resistant and conductive arteries in hypertensive patients and in experimental models of hypertension (Brown et al., 2018). Endothelial lipase knockout, which leads to elevated HDL-C levels, has been shown to attenuate vascular remodeling (Sun et al., 2012). These protective effects were attributed to HDL mediated inhibition of vascular smooth muscle cell (VSMC) proliferation and migration (Sun et al., 2012).

Angiotensin II (Ang II) signaling through the angiotensin II type 1 receptor (AT1R) stimulates hypertrophy in VSMCs from normotensive Wistar Kyoto (WKY) rats via a pathway involving extracellular signal regulated kinases 1/2 (ERK1/2) activation. This response is dependent on intracellular Ca^2+^, but independent of protein kinase C activity (El et al., 2001). In VSMCs derived from spontaneously hypertensive rats (SHR) however, Ang II-induces protein and DNA synthesis via AT1R and angiotensin II type 2 receptor (AT2R), through enhanced and sustained PI3K-dependent ERK1/2 activation (El et al., 2001). Moreover, increased Ang II–AT1R mediated phosphorylation of ERK1/2 and p38 mitogen activated protein kinase (MAPK) involved Src, a non-receptor tyrosine kinase, and is markedly enhanced in SHR (Touyz et al., 2001). This response has been linked to epidermal growth factor receptor (EGFR) transactivation and decreased activity of C-terminal Src kinase, a negative regulator of Src, in SHR-derived VSMCs (Touyz et al., 2002).

Accumulating evidence indicates a reciprocal interaction between HDL, its receptor, scavenger receptor class B type-I (SR-BI) and the renin angiotensin system (RAS) which may directly or indirectly influence various components of metabolic syndrome. We previously reported that HDL lowers blood pressure and protects spontaneously hypertensive rats (SHR) against ischemia–reperfusion injury in an SR-BI-dependent manner (Al-Jarallah and Babiker, 2022), and that these effects also involve sphingosine-1-phosphate receptors (S1PR) 1 and 3 (Al-Jarallah and Babiker, 2024). However, the precise mechanisms by which HDL reduces blood pressure in SHR remain incompletely understood.

Vascular remodeling, characterized by excessive proliferation of vascular smooth muscle cells (VSMCs), is a hallmark feature of hypertension. Enhanced signaling through the Ang II–AT1R–EGFR axis has been implicated in promoting VSMC proliferation in this context. In the present study, we hypothesized that HDL attenuates Ang II–AT1R–EGFR signaling, inhibits VSMC proliferation, and reverses aortic remodeling in SHR.

Our findings demonstrate that HDL treatment increases nitric oxide production and aortic lumen diameter, while reducing the media thickness-to-lumen diameter ratio, collagen deposition, and VSMC proliferation *in vivo*. Additionally, HDL treatment downregulates AT1R and EGFR expression and upregulates PDZK1 in SHR aortas. *In vitro*, HDL attenuates Ang II-induced VSMC proliferation by decreasing AT1R expression and suppressing Ang II-mediated EGFR transactivation. These effects are SR-BI-dependent and are reproduced by small HDL, reconstituted HDL, and lipid-free apolipoprotein A-I (Apo A-I). Together, our findings suggest that HDL-mediated inhibition of Ang II–AT1R–EGFR signaling and reversal of vascular remodeling may represent a key mechanism by which HDL lowers blood pressure in SHR.

## Materials and methods

### Materials

All materials were purchased from Sigma-Aldrich unless stated otherwise.

### Animals

Eleven-to twelve-week-old male normotensive, WKY, and hypertensive, SHR, were used in this study. Male SHR exhibit significantly higher systolic blood pressure (SBP) at 12-week of age compared to age-matched female SHR (Reckelhoff et al., 1998). Moreover, estrogen is known to modulate HDL metabolism in rodents (Parini et al., 2000) and downregulate hepatic SR-BI expression (Leiva et al., 2011). Male rats were therefore selected to avoid potential confounding effects of estrogen on HDL metabolism. Animals were housed under internationally accepted conditions in the Animal Resource Center, College of Medicine, Kuwait University with free access to food and water. All animals were kept in the same room under identical environmental conditions and were randomly assigned to the control or treatment groups. All procedures involving animals were approved by the Health Sciences Research Ethics Committee (Approval ID: 5094, Approval Date: 18-09-2016). Systolic and diastolic blood pressure (SBP and DBP) were measured in conscious animals by tail-cuff using a CODA-4-channel blood pressure system (Kent Scientific Corporation). Measurements were consistently performed at the same time of day for all groups to minimize variability due to diurnal fluctuations. SBP ≥160 mmHg was used as the threshold for hypertension. Rats that did not meet this criterion were excluded from the study.

### *In Vivo* experiments

#### Rats treatment and tissue collection

To test the effect of HDL on VSMC proliferation *in vivo*, 11-week-old WKY and SHR were randomized and treated with or without HDL, n = 7-8 rats per treatment. The selected treatment dose and duration were based on our previous study (Al-Jarallah and Babiker, 2022). Briefly, HDL (900 ng/kg/min) or PBS (as a vehicle) was continuously administered using Alzet osmotic minipumps implanted subcutaneously into the back of the rats (Al-Jarallah and Babiker, 2022). All surgical procedures were performed under aseptic conditions to minimize the risk of infection. Animals were closely monitored during recovery and postoperatively to ensure proper healing. Daily observations were made for signs of infection, swelling, or abnormal behavior. Humane endpoints, such as severe distress or significant weight loss, were predefined. No unexpected adverse events occurred, and all animals recovered successfully. After surgery, animals were placed in a recovery area with controlled temperature to maintain body heat and were monitored until they gained full consciousness. Postoperative topical analgesia (Lidocaine) was administered for 1-3 days, depending on the recovery needs of the animals. At week twelve rats were anesthetized using a combination of ketamine (50 mg/Kg, i. p.) and xylazine (5 mg/Kg, i. p.) and perfused with ice-cold phosphate-buffered saline (PBS). Thoracic aortas were collected and post-fixed in 10% neutral-buffered formalin solution for 24 h, embedded in paraffin and sectioned at 5 µm thickness.

#### Histological analysis of aortic sections

Aortic sections were deparaffinized in xylene and rehydrated through a graded series of alcohol solutions. For the evaluation of histological changes, aortic sections were stained with hematoxylin and eosin (H&E). Images were acquired using a light microscope (Axio Observer A1, Zeiss) at ×20 magnification with AxioVision software. Aortic lumen diameter (LD) and media thickness (MT) were determined using ImageJ software and compared between normotensive and hypertensive rats (Jeong et al., 2021). Elastin fibers were visualized based on their intrinsic autofluorescence using a confocal microscope at an excitation wavelength of 488 nm (Briones et al., 2003; Fritze et al., 2012). Images of elastin autofluorescence were acquired and binarized using ImageJ to measure the total area covered by elastin fibers. Collagen content was assessed using Masson’s trichrome and quantified using ImageJ software.

#### Immunofluorescence of aortic sections

Aortic sections were deparaffinized with xylene and rehydrated through a series of alcohol solutions. Antigen retrieval was performed by boiling the sections in sodium citrate buffer (10 mM, pH 6.0) for 10 min. Sections were then washed three times with phosphate buffered saline (PBS) for 7 min. Primary antibodies were prepared in 1% bovine serum albumin (BSA) PBS solution. Sections were incubated with the primary antibodies (Supplementary Table S1) overnight in a humidified chamber. The following day, sections were washed three times with PBS for 7 min and incubated with a secondary antibody tagged with Alexa Fluor 555 (1:1000; Invitrogen, CA, United States) for 2 h at room temperature. After final washes, sections were mounted and visualized under a confocal microscope (Zeiss LSM 980, Carl Zeiss, Göttingen, Germany). A series of images covering the whole area of the aortic section were acquired using 40x or ×63 objectives and stitched using ZEN imaging software (Zeiss Microscope). For analysis of PCNA, PCNA^+^ nuclei were counted using ImageJ software using the “cell counter” plug-in and expressed as a percentage of the total number of DAPI-stained nuclei. For AT1R, EGFR, SR-BI, and PDZK1, mean fluorescence intensity (MFI) was measured across the entire section and normalized to the total area.

#### Measurements of nitric oxide levels

Nitrite levels were determined in serum samples from all four treatment groups using the Griess method, as previously described (Al-Jarallah and Babiker, 2024). Briefly, serum samples or nitrite standards were mixed with 1% Griess reagent at 1:2 ratio and incubated at room temperature for 20 min. The absorbance was then measured at 540 nm. Nitrite concentrations in the samples were calculated from the standard curve and expressed relative to the total protein content in the serum.

### *In Vitro* experiments

#### Isolation of vascular smooth muscle cells

VSMCs were isolated from a separate cohort of untreated 12-week-old WKY and SHR. Thoracic aortas from four to six WKY or SHR were pooled and enzymatically digested to extract vascular smooth muscle cells (VSMCs) as previously described by us (Akhtar et al., 2012) and others (Stakos et al., 2010). Briefly, the dissected aortas were carefully cleaned of adherent adipose tissue and digested for 30 min in Dulbecco’s Modified Eagle Medium (DMEM) containing collagenase (200 U/ml), elastase (20 mg), and bovine serum albumin (BSA; 1.5 mg/mL) at 37°C. Following digestion, the adventitial tissue was peeled off and the endothelial layer was removed by gentle scraping. The remaining media layer was washed with serum-free DMEM, cut into small pieces, and incubated in the digestion mixture for 45 min at 37°C with gentle mixing in between. VSMCs were then isolated by trituration and centrifugation at 2700 rpm for 10 min. The isolated VSMCs were cultured in DMEM/F12 supplemented with 10% fetal bovine serum (FBS), 0.01% insulin transferrin sodium selenate, and 1% antibiotic-antimycotic solution. The identity of VSMCs was confirmed by immunofluorescence labeling with α-smooth muscle actin. Cells were passaged upon reaching 70%-80% confluency by trypsinization. For each experiment, the required number of cells was determined using Vi-Cell XR cell viability analyzer (Beckman Coulter). VSMCs were cultured up to *passage* 10 in this study.

#### Measurement of VSMC proliferation

VSMC proliferation was assessed using CyQUANT NF Cell Proliferation Assay Kit (Thermo Fisher, MA, United States) (Lucero et al., 2008). VSMCs were cultured in a 96-well plate at a density of 6 × 10^3^ cells per well. Cells were serum starved for 24 h then treated with HDL (100 μg/mL; Lee BioSolutions, MO, United States), for 48 h. In experiments testing the involvement of receptors, SR-BI-blocking antibody (1:500; Novus Biologicals, CO, United States) or losartan, an AT1R antagonist, (1 μM; Sigma, MO, United States), were added 1 h prior to the addition of HDL or Ang II respectively. Likewise, EGFR antagonist, AG1478 (1 μM; Abcam, MA, United States) and MEK antagonist, PD98059 (50 μM; Cell Signaling, MA, United States) were added 1 h prior to the addition of Ang II. Cell culture media was aspirated, followed by the addition of 100 µL dye binding solution for 30 min at 37°C. Fluorescence intensity was then measured using a fluorescence microplate reader (CLARIOstar, BMG LABTECH) with excitation and emission wavelengths of 485 nm and 530 nm, respectively.

#### Detection of epidermal growth factor receptor phosphorylation

Phosphorylation of the epidermal growth factor receptor (EGFR) was examined using EGFR (Phospho-Tyr1069) cell-based ELISA (Aviva System Biology, CA, United States, Cat. No.: OKAG01783). Briefly, VSMCs were cultured in a 96-well plate at a density of 6 × 10^3^ cell per well. At ∼70% confluency, the cells were serum starved for 24 h, pretreated with Ang II (10 nM) for 30 min, then treated with HDL (100 μg/mL) for 0, 5, 10, and 30 min. Cells were then fixed in 4% formaldehyde and treated with a quenching solution for 20 min. Antibodies against phospho-EGFR (Tyr1069) or total EGFR were added to the wells and incubated for 2 h at room temperature. Following washing, HRP-conjugated antibodies were added to the wells and incubated for 1.5 h at room temperature. Thereafter, cells were washed and incubated with 3,3′,5,5′-Tetramethylbenzidine substrate solution for 30 min. The stop solution was added, and the plate was read at 450 nm.

#### Immunofluorescence staining of VSMCs

VSMCs derived from WKY and SHR were cultured in 8-chamber slides for immunofluorescence staining. Following treatment, the culture media was removed, and cells were fixed with formaldehyde (3.75%) in PBS for 15 min at room temperature. Cells were then washed twice with PBS. VSMCs were blocked with 5% BSA in PBS for 1 h, followed by an overnight incubation with the primary antibody diluted in 1% BSA-PBS. The next day, the primary antibody solution was removed, and cells were washed 3 times with PBS for 5 min for each wash. The appropriate secondary antibody solution, prepared in 1% BSA-PBS, was added to cells for 1 h at room temperature. The cells were then washed three times with PBS for 5 min each and counter stained with DAPI (3 µM for 5 min). Images were captured using Zeiss Axio Imager (Zeiss LSM 700) with 20x or ×40 objectives. Fluorescent intensity of proteins of interest was normalized to either cell confluency (AT1R) or DAPI count (SR-BI, PDZK1).

### Statistical analysis

Unpaired t-test was used to compare means of two different groups when the samples followed a Gaussian distribution, as determined by the Shapiro-Wilk test. For data that did not follow a Gaussian distribution, the Wilcoxon test was applied. One-way ANOVA followed by Tukey’s *post hoc* analysis was used to compare more than two groups in the presence of one variable. Two-way ANOVA followed by Tukey’s test was employed to compare means of multiple groups in the presence of two variables. A *p-*value less than 0.05 was considered statistically significant. Data were analyzed using GraphPad Prizm software (Version No. 9.5.1) and presented as means ± standard error of the mean (SEM).

## Results

### HDL attenuates features of vascular remodeling in SHR

**FIGURE 1:**
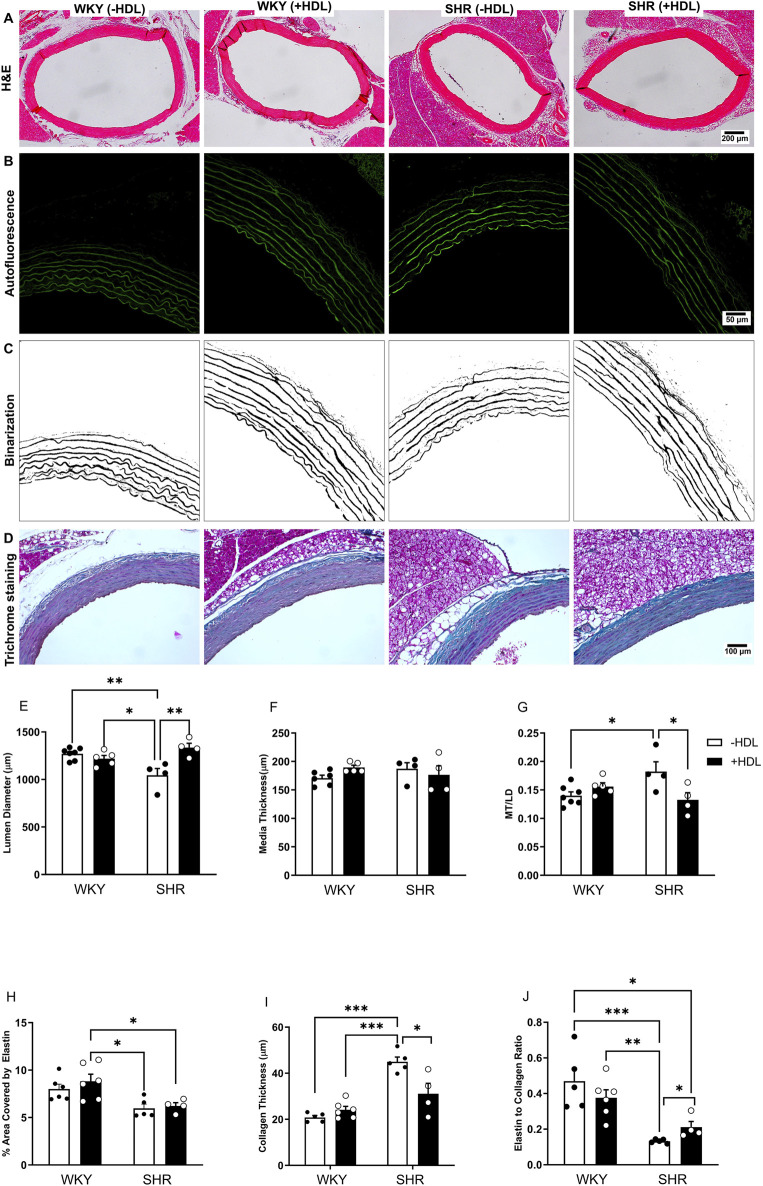
HDL treatment reverses features of vascular remodeling in SHR. Representative images of H&E-stained sections from WKY and SHR aortas treated with or without HDL **(A)**. Autofluorescent images of the elastic lamina **(B)** that were binarized **(C)** to quantify aortic elastin contents. Masson’s trichrome staining of aortic sections from vehicle or HDL-treated WKY and SHR **(D)**. Quantification of features of vascular remodeling represented as changes in lumen diameter (LD) **(E)**, media thickness (MT) **(F)**, MT to LD ratio **(G)**, elastin **(H)** collagen **(I)** contents and elastin to collagen ratio **(J)** of aortic sections from vehicle or HDL-treated WKY and SHR. Data are means ± SEM. *p < 0.05, ***p < 0.001, n = 4-7 rats per treatment.

**FIGURE 2:**
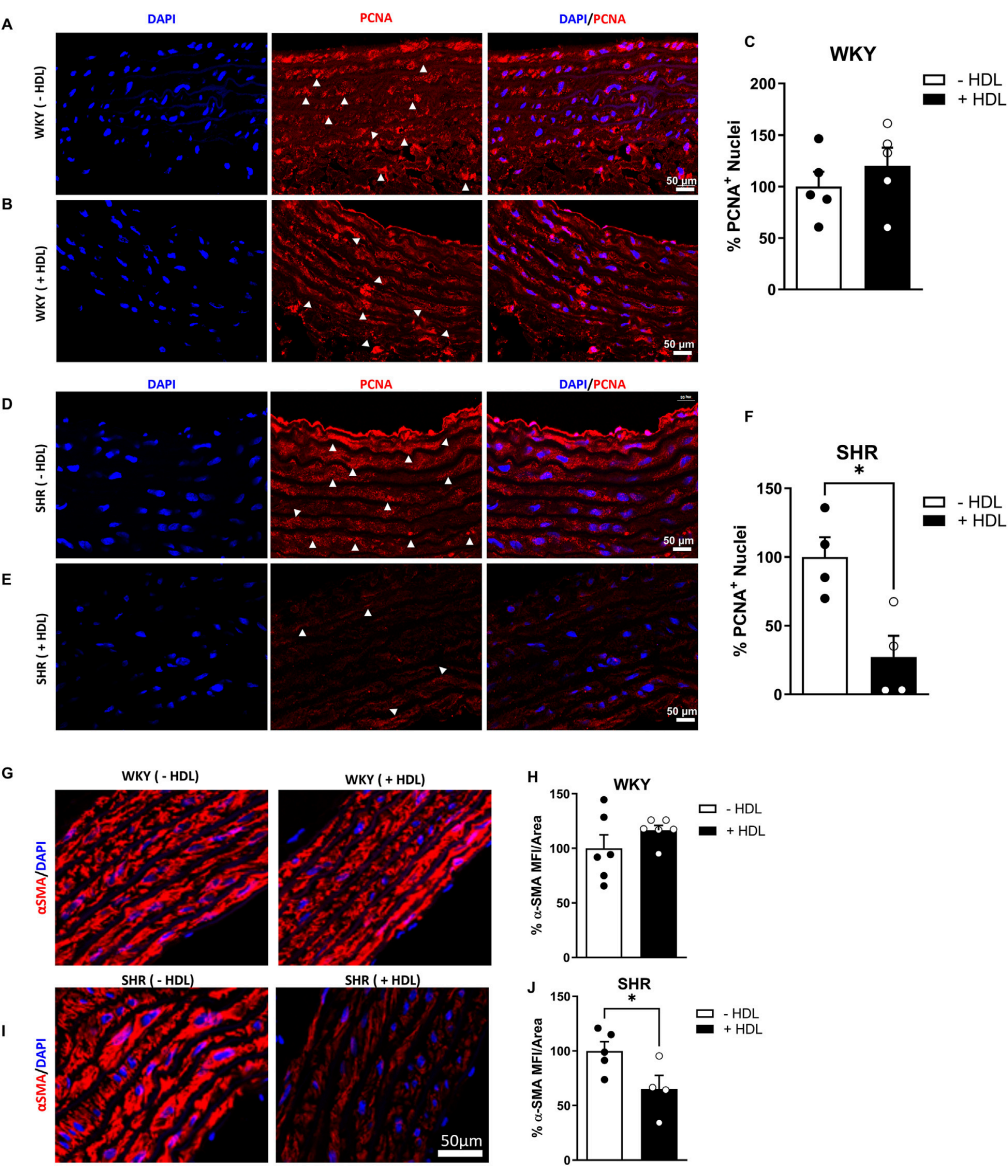
HDL reduces cellular proliferation and α-SMA expression in SHR aortas. Immunofluorescent images of proliferating (PCNA^+^, arrowheads) and DAPI stained nuclei in WKY **(A,B)** and SHR **(D,E)** aortas. Quantification the percentage of PCNA^+^ cells in vehicle-treated and HDL-treated WKY **(C)** and SHR **(F)**. α-smooth muscle actin (α-SMA) expression in aortas from vehicle and HDL-treated WKY **(G,H)** and SHR **(I,J)**. Representative immunofluorescent images of α-SMA (red) and DAPI (blue) **(G,I)** and quantification of mean fluorescent intensity (MFI) of α-SMA relative to vessel area **(H,J)**. Data are means ± SEM. Scale bar = 50 μm. *p < 0.05.

SHR exhibited significantly (p < 0.01) elevated SBP and DBP relative to WKY (Supplementary Table S2). Moreover, aortic sections from SHR demonstrated characteristic features of vascular remodeling including reduced lumen diameter (Figure 1E), increased media thickness-to-lumen diameter ratio (Figure 1G), reduced elastin content (Figure 1H), enhanced collagen deposition (Figure 1I), and a decreased elastin-to-collagen ratio (Figure 1J), relative to WKY. Interestingly, nitric oxide levels in SHR were significantly (p < 0.05) higher than in normotensive controls (Supplementary Figure S1), possibly as a compensatory response to vascular dysfunction. In addition, aortic sections from SHR expressed significantly (p < 0.05) increased percentage of proliferating (PCNA^+^) cells suggesting increased proliferation of VSMCs in these rats (Figures 2C–F). HDL treatment significantly (p < 0.05) reduced SBP and DBP in SHR without any significant effects observed in WKY (Supplementary Table S2). HDL significantly (p < 0.05) increased nitric oxide levels in HDL-treated SHR (Supplementary Figure S1). Moreover, HDL significantly (p < 0.05) increased aortic lumen diameter (Figure 1E) and decreased media thickness to lumen diameter ratio (Figure 1G). It also reduced collagen contents (Figure 1I) and increased the elastin to collagen ratio in SHR (Figure 1J). Nonetheless, HDL treatment did not affect elastin contents in SHR (Figure 1H). Finally, HDL treatment significantly (p < 0.05) reduced the percentage of proliferating cells (PCNA^+^) (Figures 2D–F) and the expression of α-SMA (Figures 2I,J) in SHR aortas compared to vehicle treated SHR. However, HDL treatment did not affect the percentage of proliferating cells (PCNA^+^) (Figures 2A–C) or α-SMA levels (Figures 2G,H) in WKY. Together these data indicate that SHR aortas exhibit multiple features of vascular remodeling that are markedly reversed by HDL treatment.

### HDL reduces the expression of AT1R and EGFR in SHR aortas

**FIGURE 3:**
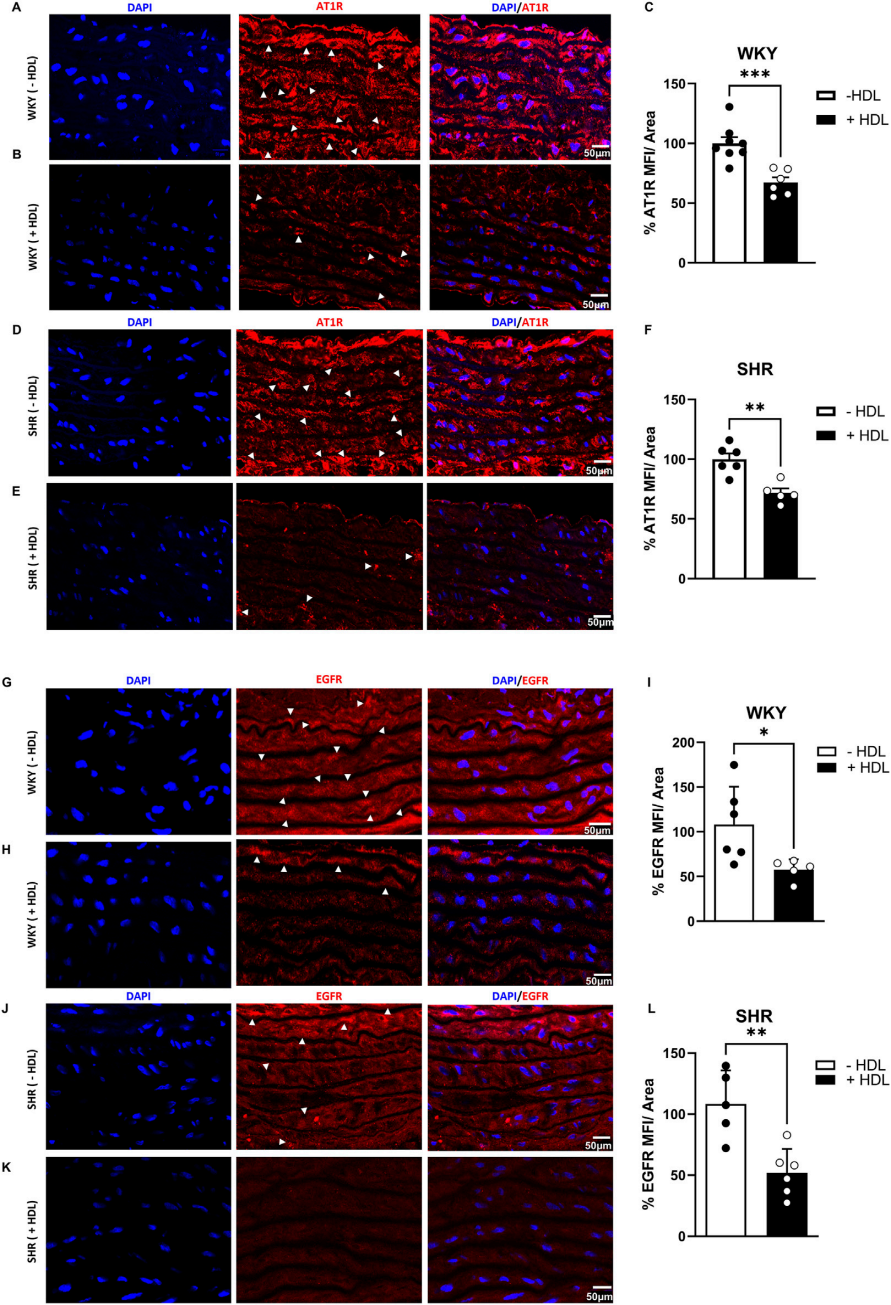
HDL reduces AT1R and EGFR expression in SHR aortas. AT1R expression in aortic sections from vehicle and HDL-treated WKY **(A–C)** and SHR **(D–F)**. Representative immunofluorescent images of AT1R (arrow heads, red) and DAPI (blue) **(A,B,D,E)** and quantification of AT1R mean fluorescent intensity (MFI) relative to tissue area **(C,F)**. EGFR expression in aortic sections from vehicle or HDL-treated WKY **(G–I)** and SHR **(J–L)**. Representative immunofluorescent images of EGFR (arrow heads, red) and DAPI (blue) **(G,H,J,K)** and quantification of EGFR mean fluorescent intensity (MFI) relative to tissue area **(I,L)**. Data are means ± SEM. Scale bar = 50 μm. *p < 0.05.

Increased VSMC proliferation has been reported to be mediated by enhanced Ang II–AT1R–EGFR–ERK1/2 signaling in SHR (Kawai et al., 2017). To test the hypothesis that HDL attenuates VSMC proliferation in SHR by negatively regulating AT1R–EGFR–ERK1/2 signaling we examined the effect of HDL treatment on AT1R, EGFR and ERK1/2 expression in aortas from rats treated with or without HDL. HDL treatment significantly (p < 0.05) reduced AT1R expression in both WKY and SHR aortas (Figures 3A–F). In addition, HDL significantly (p < 0.05) reduced aortic EGFR protein levels in SHR with a trend toward a decrease in WKY (Figures 3G–L). HDL treatment significantly (p < 0.05) increased total ERK1/2 and decreased phospho-ERK1/2 expression in WKY without any effects in aortas from SHR (Supplementary Figure S2). Together these findings suggest that HDL-mediated attenuation of AT1R–EGFR signaling may represent a mechanism by which HDL reduces VSMC proliferation *in vivo* (Figure 2).

**FIGURE 4:**
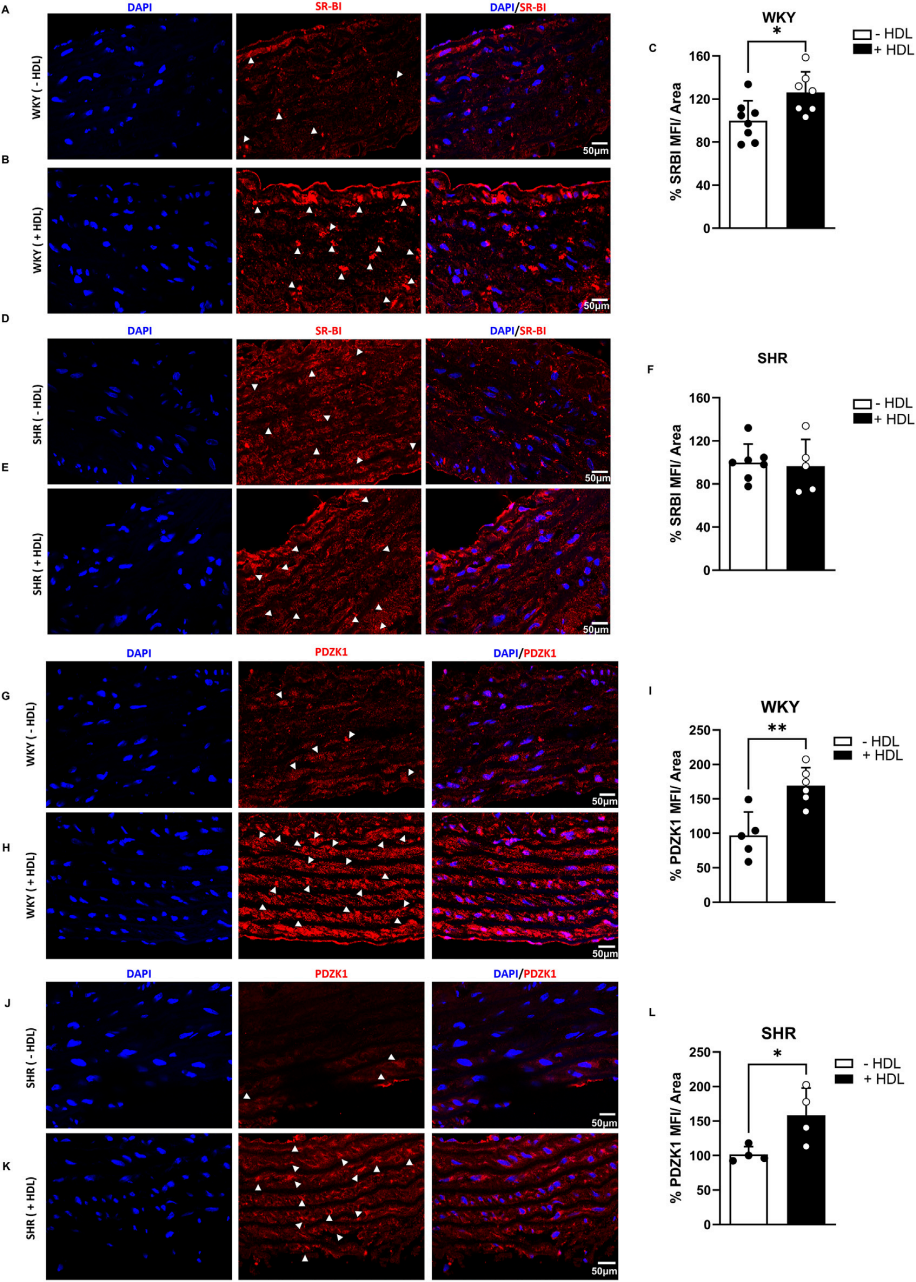
Effects of HDL on SR-BI and PDZK1 expression in WKY and SHR aortas. SR-BI expression in aortic sections from vehicle or HDL-treated WKY **(A–C)** and SHR **(D–F)**. Representative immunofluorescent images of SR-BI (arrow heads, red) and DAPI (blue) **(A,B,D,E)** and quantification of SR-BI mean fluorescent intensity (MFI) relative to tissue area **(C,F)**. PDZK1 expression in aortic sections from vehicle or HDL-treated WKY **(G–I)** and SHR **(J–L)**. Representative immunofluorescent images of PDZK1 (arrow heads, red) and DAPI (blue) **(G,K)** and quantification of PDZK1 mean fluorescent intensity (MFI) relative to tissue area **(I,L)**. Data are means ± SEM. Scale bar = 50 μm. *p < 0.05.

The HDL receptor SR-BI and its adaptor protein, PDZK1, mediate HDL signaling in the vasculature (Al-Jarallah and Trigatti, 2010). *In vivo,* HDL treatment significantly (p < 0.05) increased SR-BI (Figure 4C) and PDZK1 (Figure 4I) expression in aortas from WKY and significantly (p < 0.05) increased PDZK1 expression in aortas from SHR (Figure 4L), suggesting enhanced HDL signaling, via SR-BI and PDZK1 in aortas from HDL-treated rats.

### HDL inhibits Ang II-induced proliferation and attenuates AT1R–EGFR signaling in cultured VSMCs isolated from SHR

**FIGURE 5:**
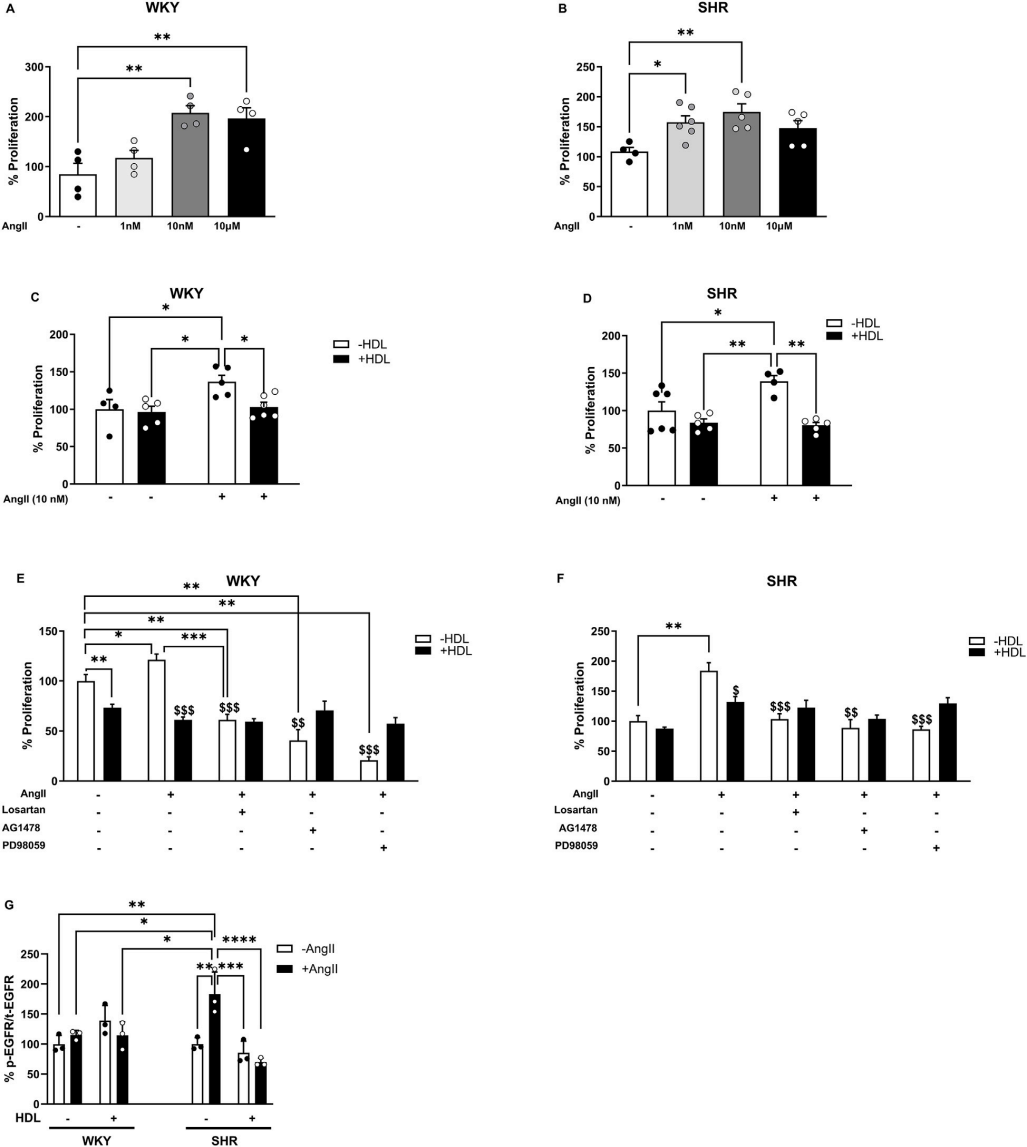
HDL reduces Ang II-induced proliferation of isolated aortic VSMCs from WKY and SHR. Proliferation of VSMCs from WKY **(A)** and SHR **(B)** treated with Ang II (1 nM, 10 nM, 10 µM) for 48 h. Effect of HDL (100 μg/mL) on Ang II (10 nM) induced proliferation of VSMCs from WKY **(C)** and SHR **(D)**. VSMCS from WKY **(E)** and SHR **(F)** were treated with Ang II (10 nM) and/or HDL (100 μg/mL) in the presence or absence of losartan (1 µM), AG147 (1 µM) or PD98059 (50 µM). Cells were pretreated with the antagonists 1 hour prior to the addition of Ang II. Ang II (10 nM) was added for 30 min then removed. Treatment with HDL and antagonists continued for 48 h and cell proliferation was measured. EGFR phosphorylation **(G)** was measured in aortic VSMCs from WKY and SHR pretreated with Ang II (10 nM) for 30 min followed by treatment with HDL (100 μg/mL) for 30 min. Data are represented as means ± SEM. *p < 0.05, **p < 0.01, ***p < 0.001, ****p < 0.0001: ^$^p < 0.05,^$$^p < 0.01,^$$$^p < 0.001 vs. (+) Ang II, (−) HDL.

To gain mechanistic insights into the interaction between HDL and the AT1R–EGFR axis in VSMC proliferation, we examined the effects of HDL on VSMCs *in vitro*. Aortic VSMCs were isolated from WKY and SHR and their smooth muscle identity was confirmed by α-SMA expression (Supplementary Figure S3). Ang II significantly (p < 0.05) increased the proliferation of VSMCs from WKY (Figure 5A) and SHR (Figure 5B) with a maximal effect observed at Ang II (10 nM) in both genotypes. HDL (100 μg/mL) significantly (p < 0.05) reduced Ang II-induced proliferation in VSMCs from WKY and SHR by 33% and 58%, respectively (Figures 5C,D).

Ang II-induced VSMCs cell growth and proliferation are mediated by AT1R, involve transactivation of EGFR, and result in enhanced phosphorylation of ERK1/2 (Kawai et al., 2017; Akhtar et al., 2016). We investigated the interaction between HDL and the Ang II–AT1R–EGFR–ERK1/2 signaling pathway in VSMC proliferation using receptor- or kinase-specific pharmacological inhibitors, as well as biochemical analysis of receptor expression and signaling molecule activation. Ang II-induced VSMC proliferation was significantly (p < 0.05) attenuated by the AT1R antagonist, losartan, the EGFR antagonist, AG1478, and the MEK/ERK1/2 inhibitor, PD98059, in VSMCs from both WKY and SHR (Figures 5E,F). Notably, HDL did not produce any additional reduction in proliferation when combined with losartan, AG1478 or PD98059. Furthermore, in VSMCs isolated from both WKY and SHR animals, the extent of HDL-induced inhibition of proliferation in the presence of losartan, AG1478 or PD98059 (in SHR only) was not significantly different from cells treated with Ang II and HDL alone (Figures 5E,F). The absence of an additive effect of HDL in the presence of receptor antagonists does not definitively confirm or exclude the involvement of AT1R or EGFR in HDL-mediated inhibition of Ang II-induced proliferation. Therefore, we further examined the effects of HDL on AT1R expression and EGFR phosphorylation. Consistent with our *in vivo* findings, HDL significantly (p < 0.05) reduced AT1R protein levels in untreated and Ang II treated VSMCs from WKY and SHR (Supplementary Figure S4) suggesting that HDL mediated downregulation of AT1R might be a key mechanism by which HDL attenuates Ang II-induced VSMC proliferation.

VSMCs from normotensive and hypertensive rats exhibited similar basal levels of phosphorylated EGFR (Figure 5G). Ang II treatment did not induce EGFR phosphorylation in WKY; however, it caused an ∼80% (p < 0.05) increase in EGFR phosphorylation in SHR-derived VSMCs (Figure 5G). Interestingly, HDL treatment significantly reduced Ang II-induced activation of EGFR (p < 0.01) (Figure 5G). Together, these data suggest that Ang II and HDL differentially regulate EGFR in VSMCs from WKY and SHR, and that HDL treatment inhibits Ang II-induced activation of EGFR in SHR (Figure 5G). These findings also indicate that HDL attenuates Ang II-induced proliferation in SHR-derived VSMCs by downregulating AT1R and suppressing AT1R-mediated transactivation of EGFR.

### HDL reduces Ang II-induced proliferation in VSMCs in an SR-BI dependent manner

**FIGURE 6:**
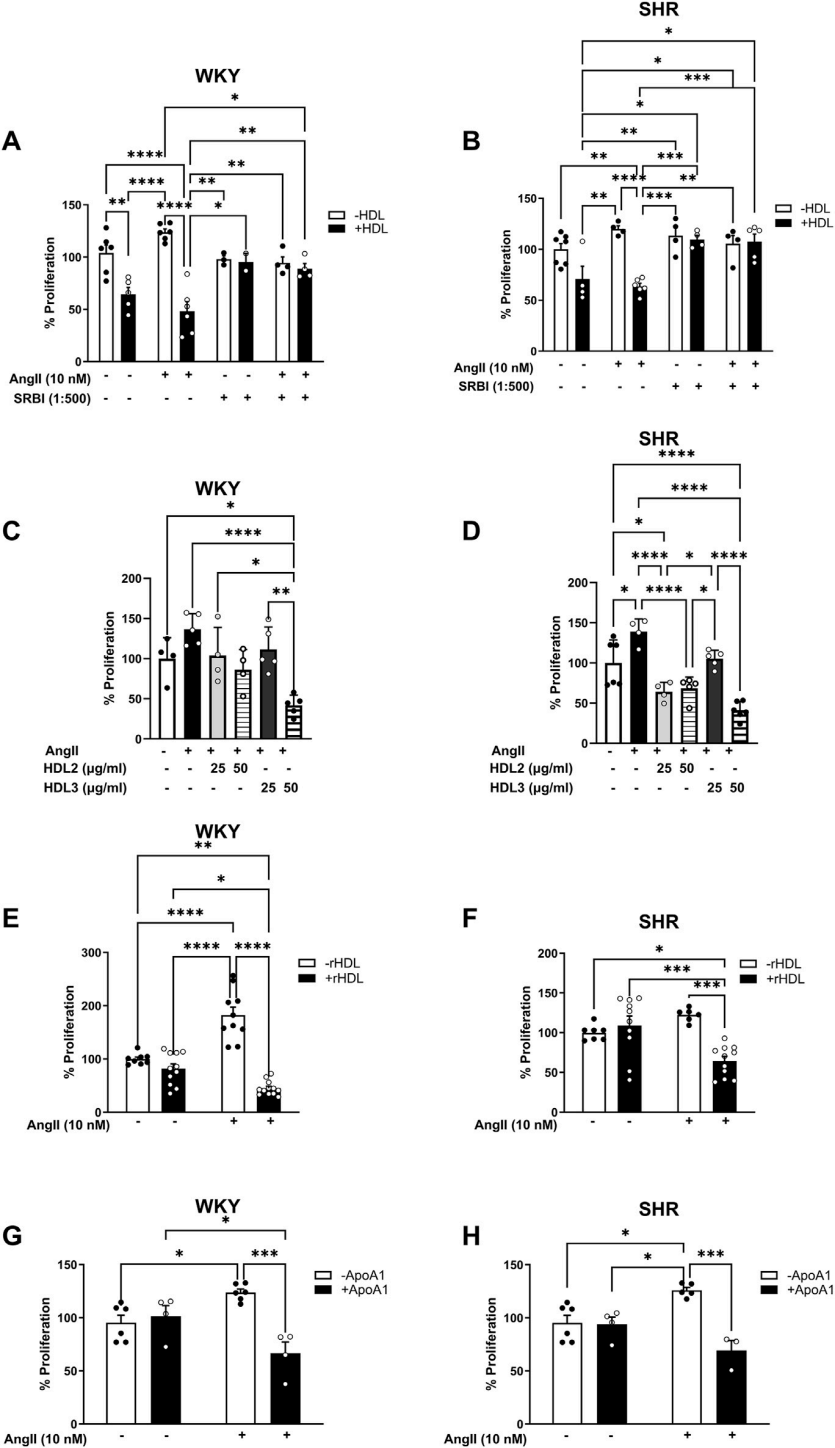
Role of SR-BI, HDL components and subpopulations in HDL-mediated inhibition of VSMC proliferation. VSMCs from WKY **(A)** and SHR **(B)** were pretreated with Ang II (10 nM) for 30 min, followed 1 h incubation with SR-BI blocking antibody (1:500). HDL (100 μg/mL) was then added in the presence of SR-BI blocking antibody for 48 h and proliferation was measured. Alternatively, VSMCs from WKY and SHR were treated with Ang II (10 nM) followed by HDL2 (25, 50 μg/mL), HDL3 (25, 50 μg/mL) **(C,D)**, rHDL (50 μg/mL) **(E,F)** or Apo A-I (100 μg/mL) **(G,H)** for 48 h and cell proliferation was measured. Data are means ± SEM. *p < 0.05, **p < 0.01, ***p < 0.001, ****p < 0.0001.

The requirement of SR-BI in HDL-mediated reduction of Ang II-induced VSMC proliferation was tested using an SR-BI blocking antibody (Figure 6). The presence of SR-BI blocking antibody significantly diminished the antiproliferative effects of HDL in VSMCs from WKY (p < 0.01) and SHR (p < 0.001) (Figures 6A,B), indicating that HDL must bind to SR-BI to exert its inhibitory effects. Additionally, HDL treatment (100 μg/mL for 48 h) significantly (p < 0.05) increased the expression levels of SR-BI and PDZK1in VSMCs from normotensive and hypertensive rats, regardless of Ang II treatment (Supplementary Figure S5). This suggests that HDL upregulates SR-BI and PDZK1 expression, further supporting its role in modulating VSMC proliferation through SR-BI-dependent pathways. These findings demonstrate HDL’s ability to attenuate Ang II-induced VSMC proliferation requires SR-BI and is associated with increased expression of SR-BI and its adaptor protein PDZK1.

HDL represents a heterogeneous group of particles that differ in size, shape, and composition. Different HDL subpopulations have been shown to exert distinct functions (Cho, 2009; Davidson and Thompson, 2007; Davidsson et al., 2009; Heinecke, 2009; Movva and Rader, 2008; Tabet and Rye, 2009). To test whether the observed inhibitory effect of HDL on Ang II-induced proliferation is specific to a particular HDL subpopulation, we examined the effects of large (HDL2) and small (HDL3) HDL particles on Ang II-induced proliferation (Figures 6C,D). HDL2 (50 μg/mL) and HDL3 (50 μg/mL) significantly (p < 0.01) reduced Ang II-induced proliferation in VSMCs isolated from WKY. However, this was not observed at a lower dose (25 μg/mL) of either subpopulation in these cells. In VSMCs from SHR, by contrast, both HDL2 and HDL3 significantly (p < 0.01) reduced Ang II-induced proliferation at both doses tested (25 and 50 μg/mL) (Figures 6C,D). These data suggest that both large and small HDL particles can reduce Ang II-induced proliferation of VSMCs isolated from normotensive and hypertensive rats. Moreover, HDL carries a wide range of bioactive molecules that mediate its protective actions (Cho, 2009; Davidson and Thompson, 2007; Davidsson et al., 2009; Heinecke, 2009; Movva and Rader, 2008; Tabet and Rye, 2009). To test whether the simplest form of HDL is sufficient to inhibit Ang II effects on VSMC proliferation, we used HDL particles reconstituted from phosphatidylserine and Apo A-I (reconstituted HDL, rHDL). Treatment with rHDL significantly (p < 0.001) reduced Ang II-induced proliferation of VSMCs from both WKY and SHR suggesting that rHDL is sufficient to attenuate the proliferative effects of Ang II in VSMCs (Figures 6E,F). Finally, to distinguish between the requirement of the HDL particle versus lipid free apolipoprotein, we examined the effect of lipid free Apo A-I (Figures 6G,H). Apo A-I significantly (p < 0.05) reduced Ang II-induced proliferation in VSMCs from WKY and SHR, indicating that lipid-free Apo A-I alone is sufficient to inhibit Ang II effects (Figures 6G,H). Collectively, these data suggest the requirement of SR-BI in mediating the antiproliferative actions of HDL. In addition, they imply that the antiproliferative effects of HDL are not restricted to a particular HDL subpopulation, but can be mediated by small, large, reconstituted particles or lipid-free Apo A-I.

## Discussion

**FIGURE 7:**
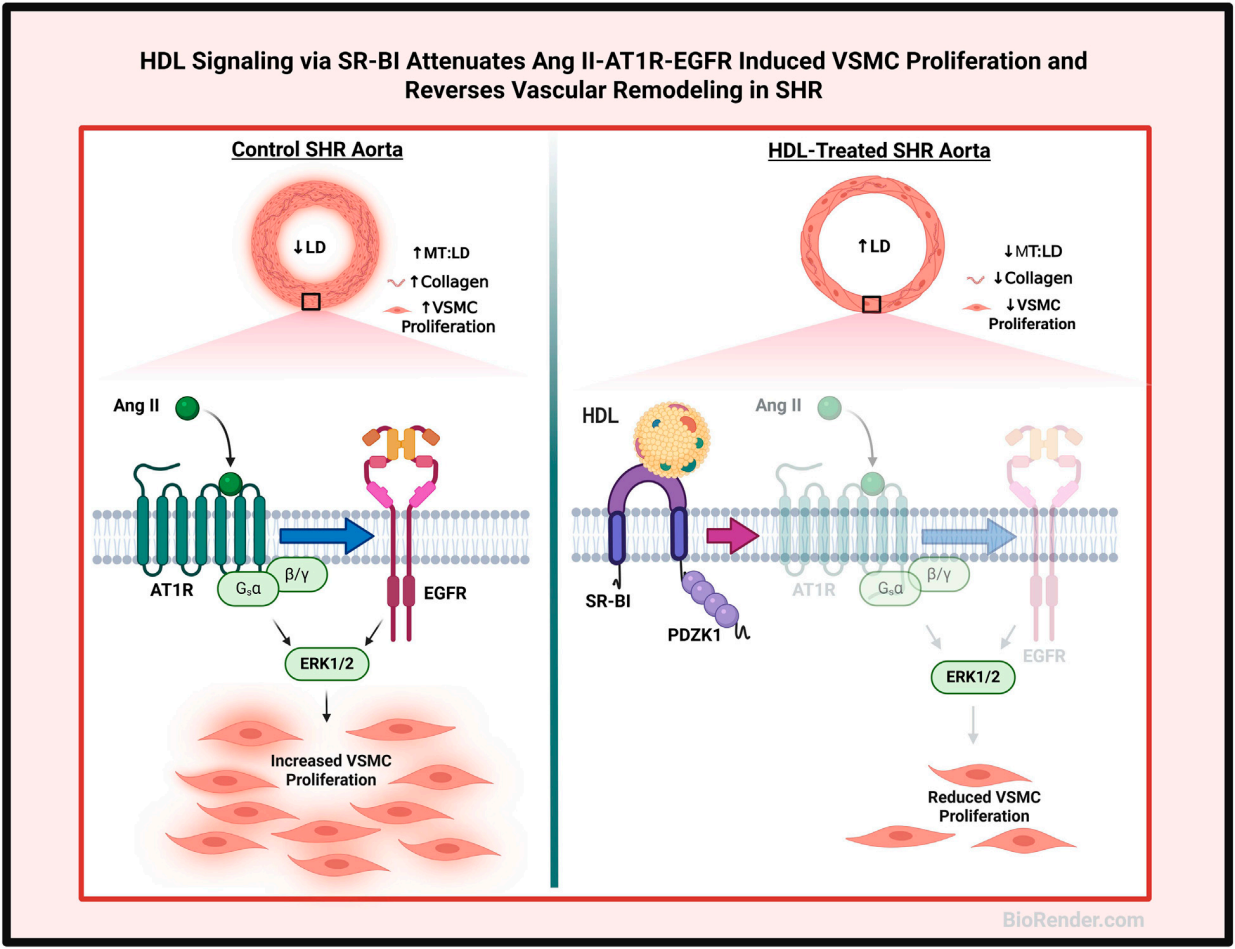
Proposed mechanism of HDL effects on vascular remodeling. Aortas from hypertensive rats exhibited narrower lumen, thicker media and increased collagen contents and number of proliferating VSMCs relative to aortas from normotensive rats. HDL treatment increased lumen diameter (LD), decreased media thickness (MT), and reduced collagen contents and VSMC proliferation in aortas from hypertensive rats. HDL signaling via SR-BI, downregulation of AT1R and EGFR expression and inhibition of Ang II-induced transactivation of EGFR inhibited the proliferation of VSMCs from hypertensive rats. Created using Biorender.com.

In this study we identify a novel role for HDL in modulating vascular remodeling in hypertensive rats. We demonstrate that HDL treatment increases aortic lumen diameter, reduces media thickness to lumen diameter ratio and diminishes collagen contents in SHR aortas. Moreover, HDL treatment decreases the percentage of actively proliferating VSMCs and α-SMA protein expression in SHR aortas. HDL treatment decreases AT1R and EGFR expression and increases the expression levels of SR-BI adaptor protein, PDZK1, in aortic sections from SHR. Mechanistically we report that HDL attenuates Ang II-induced VSMC proliferation by reducing AT1R protein levels and inhibiting the activation of EGFR. These effects are mediated by SR-BI and are reproduced by large, small, reconstituted HDL particles and lipid free Apo A-I (Figure 7).

Hypertension induced changes in the vasculature including increased aortic wall thickness, elevated collagen content and reduced vessel diameter, are well documented structural alterations in SHR (Burla et al., 2007; Stauss et al., 2011). Antihypertensive medications have been shown to reduce blood pressure and reverse features of vascular remodeling in SHR (Burla et al., 2007; Fernandes-Santos et al., 2009). In this study we report that HDL administration lowers SBP and DBP, increases nitric oxide levels, and significantly alters the vascular architecture in SHR. HDL-treated SHR exhibited increased aortic lumen diameter, reduced media thickness-to-lumen diameter ratio, diminished collagen deposition, and elevated nitric oxide levels (Figure 1). Additionally, HDL treatment decreased the number of actively proliferating VSMCs and reduced α-SMA expression in hypertensive rat aortas (Figure 2). These findings suggest a protective role for HDL in attenuating hypertension-induced vascular remodeling and may provide a mechanistic link to its antihypertensive effects.

The HDL-induced structural alterations in the vessel wall may enhance responsiveness to endogenous vasodilators or reduce sensitivity to vasoconstrictors in SHR. Previous studies support HDL-induced vasorelaxation in rodent aortas (Yuhanna et al., 2001) and report a positive correlation between HDL-C and Apo A-I levels and arterial dilation in humans (Ferre et al., 2011). HDL from healthy normolipidemic individuals reversed the inhibitory effect of oxidized low-density lipoprotein (oxLDL) on arterial relaxation (Matsuda et al., 1993). Small dense HDL particles exhibited a more potent nitric oxide-dependent vasorelaxant effect as compared to larger particles which was attributed to their enrichment with sphingosine-1-phosphate (Persegol et al., 2018). Conversely, HDL from abdominally obese patients (Persegol et al., 2007a) and patients with type I (Persegol et al., 2007b) or type II (Persegol et al., 2006) diabetes mellitus failed to counteract the inhibitory effect of oxLDL on vasorelaxation suggesting impairments in HDL function in these individuals. Interestingly these impairments in HDL vasorelaxant functions were independent of HDL-C levels as raising HDL-C with glitazones did not restore HDL-mediated vascular relaxation (Persegol et al., 2014). Importantly, we did not observe an increase in HDL-C levels following HDL treatment in SHR or WK (Al-Jarallah and Babiker, 2022). We therefore speculate that HDL-mediated protection against myocardial ischemia/reperfusion injury (Al-Jarallah and Babiker, 2022) and HDL-induced reversal of vascular remodeling are independent of changes in HDL-C levels and may be attributed to restored HDL functionality.

Treatment of SHR with losartan reversed vascular remodeling via a mechanism that involved downregulation of p22 (phox) and inhibition of oxidative stress (Li et al., 2010). Similarly, 6-week treatment of SHR with eplerenone, an aldosterone receptor antagonist, reduced blood pressure and attenuated cardiovascular remodeling in these rats (Burla et al., 2007). Moreover, hypertension induced structural changes in the vasculature were reversed by rosuvastatin via its anti-inflammatory and anti-apoptotic effects (Lv et al., 2020). Long term treatment of SHR with olmesartan reduced blood pressure and reversed some features of hypertension induced vascular remodeling. Reciprocal interaction of olmesartan with AT1R and AT2R components of RAS was suggested as a possible mechanism for the observed protective effects (Fernandes-Santos et al., 2009). Our data provide compiling evidence that HDL mimics the effects of antihypertensive medications by lowering blood pressure and reversing vascular remodeling, thereby highlighting a novel therapeutic role for HDL in hypertension management. HDL-mediated inhibition of Ang II–AT1R–EGFR signaling might represent a mechanism by which HDL exerts its vasculoprotective and anti-hypertensive effects. While these finding support the concept of targeting vascular remodeling in hypertension, relatively few studies have directly investigated the antihypertensive actions of HDL. Our results complement and expand upon existing literature by showing that HDL not only reduces blood pressure (Al-Jarallah and Babiker, 2022) but also reverses vascular remodeling in SHR. This aligns with previous reports demonstrating HDL-mediated improvements in endothelial function and nitric oxide bioavailability (Mineo et al., 2003), as well as suppression of the renin-angiotensin system (Van Linthout et al., 2009; Lin et al., 2015).

Enhanced VSMC proliferation is a hallmark feature of vascular remodeling in hypertension (Brown et al., 2018). We therefore examined the effects of HDL on Ang II-induced proliferation of VSMCs isolated from WKY and SHR. Ang II-induced proliferation was mediated through AT1R–EGFR–ERK1/2 signaling, as receptor/kinase antagonist effectively blocked this proliferation (Figure 5). Ang II treatment, however, did not induce EGFR phosphorylation in VSMCs from WKY, possibly because the optimal time window for detecting phosphorylation was missed during the tested time points. HDL treatment, however, inhibited Ang II-mediated activation of EGFR in VSMCs from SHR. Consistent with our findings, HDL treatment prevented oxLDL-induced activation of EGFR and subsequent matrix metallopproteinase-2 activation in VSMCs, an effect attributed to HDL’s antioxidant properties (Robbesyn et al., 2005). In contrast to native HDL, oxidized HDL increased VSMC proliferation and migration of by promoting reactive oxygen species generation (Wang et al., 2014). The involvement of other functional mechanisms of HDL, however, cannot be excluded. For instance, cholesterol depletion by cholesterol binding agents such as cyclodextrin or rosuvastatin has been shown to alter AT1R structure, reduce its binding affinity to Ang II, and decrease the expression of functional AT1R (Matsuo et al., 2019). Therefore, HDL-mediated cholesterol efflux from VSMCs may represent an additional mechanism through which HDL blocks Ang II-induced transactivation of EGFR in SHR-derived VSMCs. In line with our data, HDL was previously shown to reduce EGF-induced DNA synthesis in VSMCs, an effect that was reproduced by purified Apo A-I and Apo A-II (Ko et al., 1993). Available evidence indicates reduced HDL-C levels in hypertensive rodents (Al-Jarallah and Babiker, 2022) and patients (Wu et al., 2022) supporting hypertension-induced HDL dysfunction (Ross et al., 2015). Dysfunctional HDL, generated *in vitro* by glycation or *in vivo* from diabetic patients, induced VSMC proliferation, migration, and reactive oxygen species generation (Du et al., 2017). Thus, the observed protective effects of HDL in SHR could be attributed to restored HDL composition and/or improved function.

HDL treatment reduced AT1R expression in VSMCs from SHR in the presence and absence of Ang II (Supplementary Figure S4). This is consistent with our *in vivo* findings that HDL inhibited aortic VSMC proliferation and reduced AT1R and EGFR receptor expression in hypertensive rats (Figure 3). These observations suggest the existence of at least two mechanisms by which HDL reduces VSMC proliferation in hypertensive rats: inhibition of Ang II-mediated activation of EGFR and downregulation of AT1R expression in these cells. Analogous modulation of AT1R expression by HDL has been reported in other cell types under different pathological stimuli. HDL reduced hyperglycemia-induced AT1R expression in human aortic endothelial cells (Van Linthout et al., 2009), and also reduced mechanical stress-induced AT1R expression in cultured cardiomyocytes and in mouse hearts (Lin et al., 2015). Therefore, HDL-induced alterations in AT1R expression in other vascular cells in SHR cannot be excluded as additional mechanisms through which HDL reverses vascular remodeling and attenuates hypertension via its interaction with the renin-angiotensin system. Furthermore, HDL protective effects in other vascular beds could contribute to the observed reductions in blood pressure. Interestingly, serum EGF levels were positively correlated with diastolic blood pressure and arterial stiffness, and negatively correlated with serum HDL, Apo A-I, and Apo A-II levels in a healthy population (Lundstam et al., 2007). HDL reduced EGF-induced DNA synthesis in VSMCs in a dose-dependent manner (Ko et al., 1993). These effects were mimicked by HDL’s protein faction and by purified Apo A-I and Apo A-II (Ko et al., 1993), suggesting that HDL may exert direct inhibitory effects on EGFR signaling, independent of its blockade of Ang II-induced EGFR transactivation. Moreover, potential interactions between HDL and other mediators of VSMC proliferation in hypertension, such as endothelin and aldosterone, cannot be excluded. HDL may attenuate Ang II-induced VSMC proliferation by acting at the receptor level by reducing the expression of AT1R and EGFR and/or blocking AT1R-mediated transactivation of EGFR. Alternatively, HDL might inhibit Ang II-induced proliferation by reducing Ang II synthesis; however, this possibility was excluded, as HDL treatment did not alter Ang II levels in SHR aortas (data not shown).

In addition to downregulating AT1R and EGFR, HDL increased SR-BI (*in vitro*) and PDZK1 (*in vitro and in vivo*) protein levels in SHR, suggesting enhanced SR-BI-mediated signaling in response to HDL. SR-BI and its adaptor protein, PDZK1, mediated the protective actions of HDL in the vasculature (Al-Jarallah and Trigatti, 2010). SR-BI activation of endothelial nitric oxide synthase increased nitric oxide production and relaxed rat aortas form wild type mice (Yuhanna et al., 2001). Independent of SR-BI, PDZK1 inhibited VSMC proliferation and prevented neointima formation via interaction with break point cluster kinase (Lee et al., 2015). This suggests the possibility that in SHR, HDL-induced protection via increased PDZK1 expression may be independent of an increase in SR-BI expression levels. This interpretation aligns with the lack of *in vivo* increase in SR-BI expression in HDL-treated SHR, despite observed alterations in vascular structure. The differential regulation of SR-BI and PDZK1 observed *in vivo* (Figure 4) versus *in vitro* (Supplementary Figure S5) reflects critical tissue-specific and microenvironmental influences on HDL signaling. In hypertensive rats, systemic inflammation, and oxidative stress likely impar SR-BI upregulation despite HDL administration, whereas isolated VSMCs retain responsiveness to HDL, showing 2–3-fold increases in both SR-BI and PDZK1 expression. These findings highlight the importance of microenvironmental context and caution against extrapolating cell culture results into intact organisms without validation.

Our study provides a mechanistic understanding of HDL-induced desensitization of Angiotensin II–AT1R–EGFR signaling and vascular remodeling in hypertensive rats, thereby supporting its potential therapeutic application in hypertension. The strengths of our work include the use of both *in vivo* and *in vitro* approaches utilizing a well-characterized rodent model of hypertension. Nonetheless, there are some shortcomings. These include the short duration of HDL treatment, the exclusive use of male rats, reliance on semi-quantitative protein analysis methods, and the lack of functional assays on vascular reactivity. Despite the significance of the present work in identifying mechanisms underlying the anti-hypertensive effects of HDL, future studies should incorporate long-term treatment protocols, include female rat, and assess functional vascular outcomes.

Vascular remodeling is a multifactorial process involving mediators such as endothelin-1, nitric oxide and oxidative stress. Our study indicates that HDL increases nitric oxide levels in SHR. This is consistent with extensive evidence demonstrating that HDL, via SR-BI, stimulates endothelial nitric oxide synthase-mediated production of nitric oxide thereby inducing vasodilatation (Mineo et al., 2003; Riwa et al., 2013). In addition, HDL exhibits antioxidant properties which may mitigate oxidative stress, a key driver of vascular injury in hypertension (Riwa et al., 2013; Sorrentino et al., 2010). Clinical studies associated improved HDL levels with lower circulating endothelin-1 and better endothelial function (Kuang et al., 2025), while experimental evidence indicated that HDL directly stimulates endothelin-1 production in endothelial cells (Hu et al., 1994), reflecting both direct cellular and systemic effects. The levels of endothelin-1 in HDL-treated SHR, therefore, warrant further investigation. Moreover, HDL inhibited cytokine-induced proliferation of VSMCs (van et al., 2013), stimulated the migration of macrophages (Al-Jarallah et al., 2014) and enhanced the proliferation of endothelial cells (Jin et al., 2018). Collectively, these findings suggest the presence of multiple vasoprotective mechanisms through which HDL may modulate vascular structure and function, ultimately attenuating hypertension.

## Conclusion

In conclusion, HDL treatment reversed hypertension-induced aortic remodeling in SHR. HDL altered the geometric dimensions of the thoracic aorta by increasing aortic lumen diameter and reducing media thickness to lumen diameter ratio. In parallel, HDL altered the biochemical features of the aorta, as evidenced by reduced collagen contents, decreased cellular proliferation and expression of genes implicated in enhanced VSMC proliferation. Our data suggest that HDL attenuates Ang II–AT1R–EGFR signaling and inhibits VSMC proliferation, supporting the therapeutic potential of HDL in mitigating vascular remodeling and lowering blood pressure in hypertension (Figure 7).

## Data Availability

The original contributions presented in the study are included in the article/Supplementary Material, further inquiries can be directed to the corresponding author.

## Supplementary material

SUPPLEMENTARY FIGURE 1 HDL increases nitric oxide levels in SHR. Serum samples from vehicle or HDL-treated WKY and SHR were assayed for nitrite, as a measure of nitric oxide. Data are means ± SEM. *p < 0.05, ****p < 0.0001.

SUPPLEMENTARY FIGURE 2 The effect of HDL on total and phospho-ERK1/2 expression in WKY and SHR aortas. Total ERK1/2 expression in aortic sections from vehicle or HDL-treated WKY (A-C) and SHR (D-F). Representative immunofluorescent images of total ERK1/2 (arrow heads, red) and DAPI (blue) (A, B, D, E) and quantification of ERK1/2 mean fluorescent intensity (MFI) relative to tissue area (C, F). Phospho-ERK1/2 expression in aortic sections from vehicle or HDL-treated WKY (G-I) and SHR (J-L). Representative immunofluorescent images of total ERK1/2 (arrow heads, red) and DAPI (blue) (G, H, J, K) and quantification of ERK1/2 mean fluorescent intensity (MFI) relative to tissue area (I, L). Data are means ± SEM. Scale bar = 50 μm. *p < 0.05.

SUPPLEMENTARY FIGURE 3 Characterization of aortic VSMCs isolated from WKY and SHR. Immunofluorescence staining of α-smooth muscle actin (green) and nuclei (blue) in VSMCs.

SUPPLEMENTARY FIGURE 4 HDL reduces AT1R expression in VSMCs from WKY and SHR rats. VSMCs from WKY (A) and SHR (B) were labeled with AT1R (green) and counterstained with DAPI (blue). The mean fluorescent intensity (MFI) of AT1R was normalized to cell confluency. VSMCs were pretreated with Ang II (10 nM) for 30 min, followed by HDL (100 μg/mL) for 48 h. Data are means ± SEM. **p < 0.01, ***p < 0.001.

SUPPLEMENTARY FIGURE 5 HDL increases SR-BI and PDZK1 expression in VSMCs from WKY and SHR. VSMCs from WKY (A) and SHR (B) were labeled with SR-BI (green) and counterstained with DAPI (blue). The mean fluorescent intensity (MFI) of SR-BI was normalized to DAPI number. Data are presented as means ± SEM. *p < 0.05, **p < 0.01.

## Abbreviations

VSMCs, Vascular smooth muscle cell; Ang II, angiotensin II; AT1R, angiotensin II type 1 receptor; EGFR, epidermal growth factor receptor; SHR, spontaneously hypertensive rats; WKY, Wistar Koyoto rats; HDL, high density lipoprotein; SR-BI, scavenger receptor class B type-I; PSD-95, postsynaptic density protein; dlg, *Drosophila* discs-large; PDZK1, tight-junction protein, ZO1; ERK1/2, extracellular signal regulated kinases 1/2; CHD, coronary heart disease; HDL-C, HDL cholesterol; Apo A-I, apolipoprotein A-I; LD, aortic lumen diameter; MT, media thickness; PBS, phosphate buffered saline; BSA, bovine serum albumin; DMEM, Dulbecco’s Modified Eagle Medium; rHDL, reconstituted HDL; oxLDL, oxidized low density lipoprotein.
