# Supplementary figures and images for: HDL attenuates Ang II–AT1R–EGFR signaling and reverses vascular remodeling in spontaneously hypertensive rats
Source: Front Pharmacol. 2025 Jul 29;16:1617420. doi: 10.3389/fphar.2025.1617420 (PMC12339555; doi:10.3389/fphar.2025.1617420)

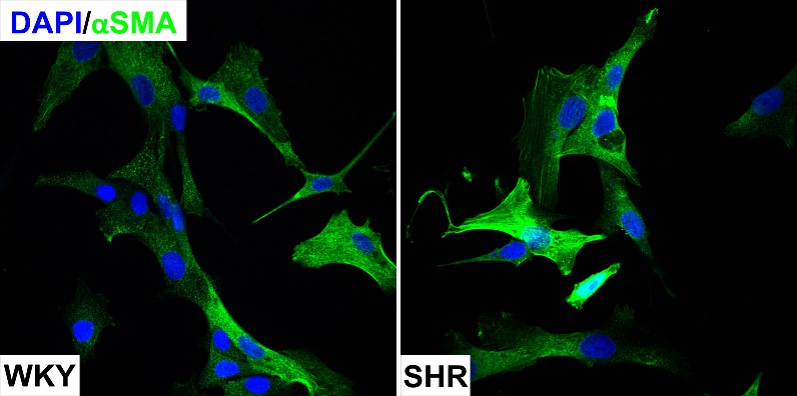

Supplement: Supplementary file 1 [file Image3.jpeg]

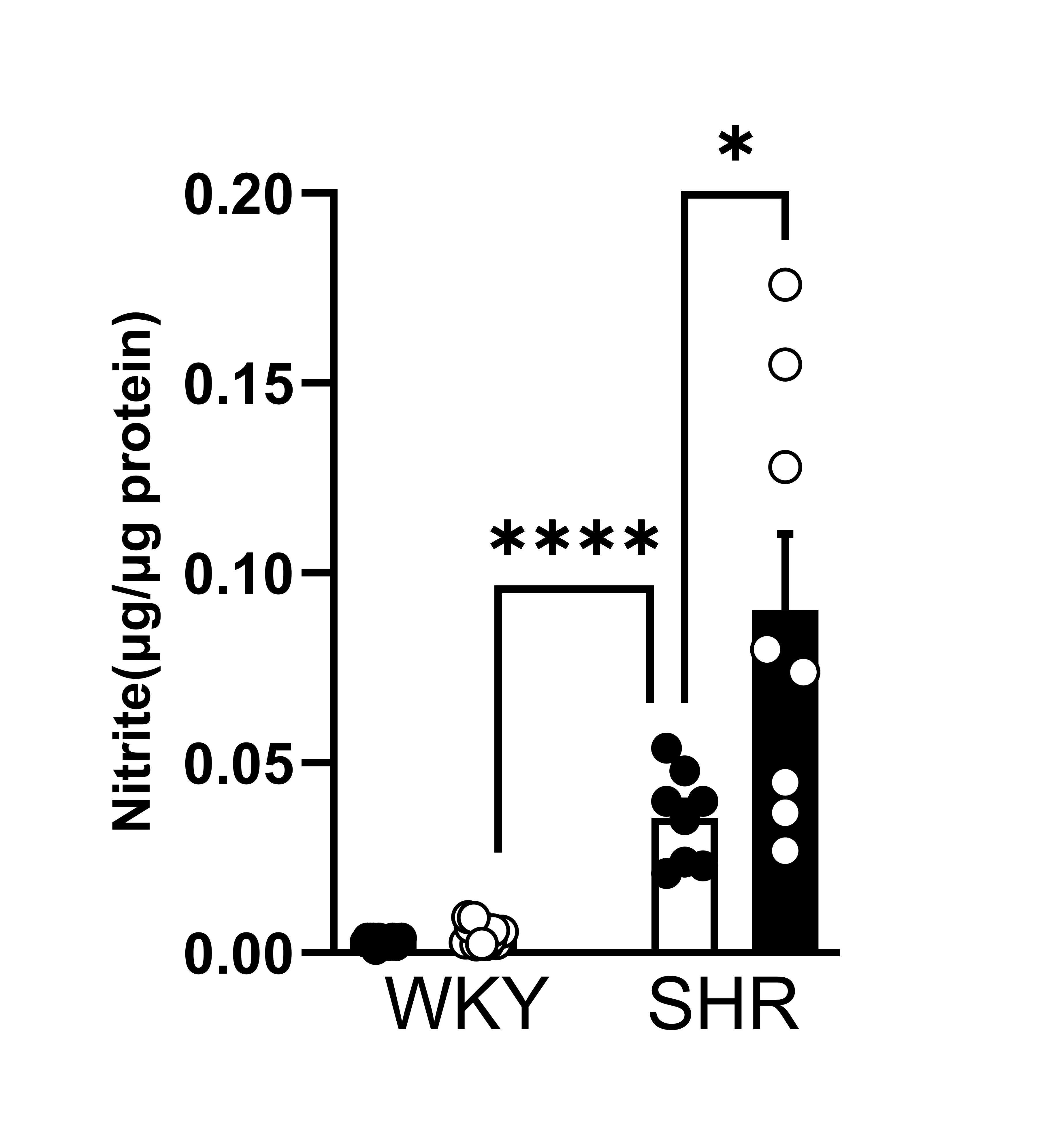

Supplement: Supplementary file 3 [file Image1.jpeg]

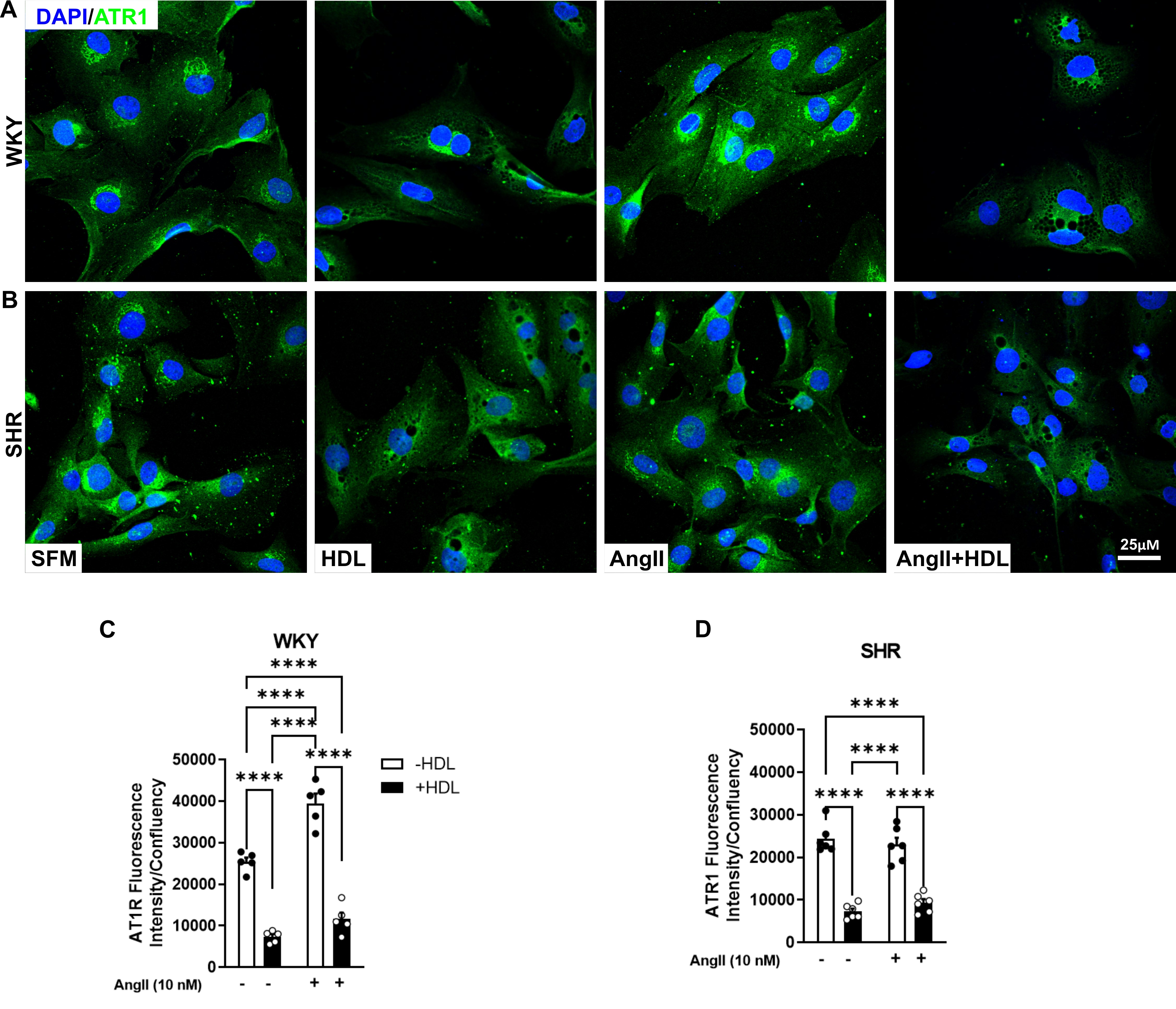

Supplement: Supplementary file 4 [file Image4.jpeg]

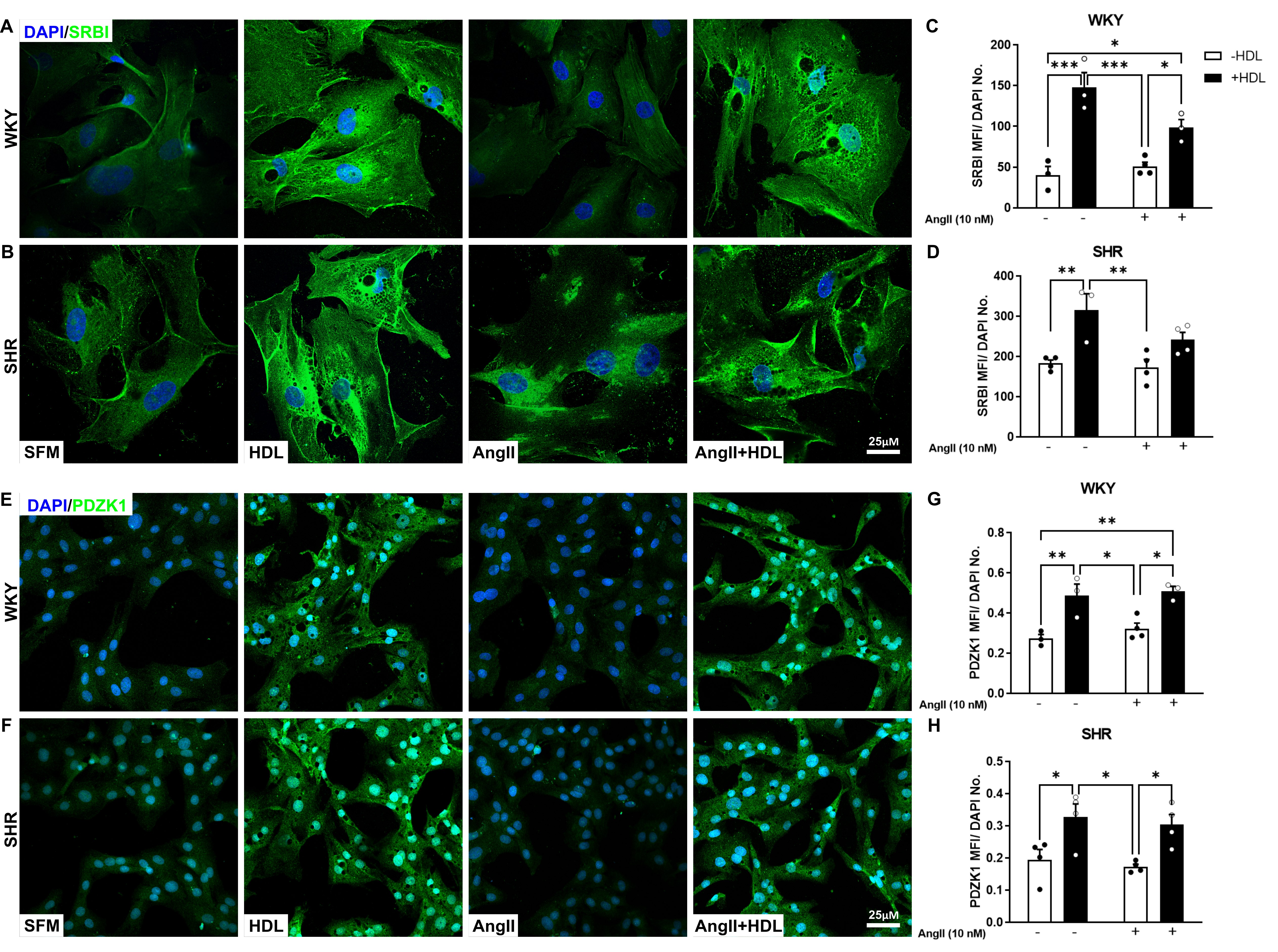

Supplement: Supplementary file 6 [file Image5.jpeg]
