# Supplementary material for: HDL attenuates Ang II–AT1R–EGFR signaling and reverses vascular remodeling in spontaneously hypertensive rats
Source: Front Pharmacol. 2025 Jul 29;16:1617420. doi: 10.3389/fphar.2025.1617420 (PMC12339555; doi:10.3389/fphar.2025.1617420)
Supplement: Supplementary file 2 [file Supplementaryfile1.docx]

**Supplementary Table-1: Antibodies used in immunofluorescence staining of aortic sections from WKY an SHR treated with or without HDL.**

| **Primary antibodies** | **Dilution** | **Company** | **Secondary antibodies** |
| --- | --- | --- | --- |
| **Monoclonal mouse monoclonal anti-PCNA** | **1:5000** | **Abcam, MA, USA** | **Alexa Fluor 555 donkey**  **anti-mouse IgG** |
| **Rabbit anti-α-SMA** | **1:10,000** | **Abcam, MA, USA** | **Alexa Fluor 555 donkey**  **anti-rabbit IgG** |
| **Rabbit anti-ATR1** | **1:200** | **Genetex, CA, USA** | **Alexa Fluor 555 donkey**  **anti-rabbit IgG** |
| **Rabbit anti-EGFR** | **1:200** | **Sigma, MO, USA** | **Alexa Fluor 555 donkey**  **anti-rabbit IgG** |
| **Rabbit anti-ERK1/2** | **1:200** | **Cell Signaling, MA, USA** | **Alexa Fluor 555 donkey**  **anti-rabbit IgG** |
| **Rabbit anti-phospho-ERK1/2** | **1:200** | **Cell Signaling, MA, USA** | **Alexa Fluor 555 donkey**  **anti-rabbit IgG** |
| **Rabbit anti-SRBI** | **1:500** | **Novus Biologicals, CO, USA** | **Alexa Fluor 555 donkey**  **anti-rabbit IgG** |
| **Rabbit anti-PDZKI** | **1:400** | **Abcam, MA, USA** | **Alexa Fluor 555 donkey**  **anti-rabbit IgG** |

**Supplementary Table-2: Blood pressure readings of WKY and SHR pre- and post- HDL treatment.**

^#^p < 0.05 vs WKY, ^*^p < 0.05 vs pre-implantation, ^$^p < 0.05 vs control SHR, n=7 rats per group.

|  | **Control** | | | | **HDL** | | | |
| --- | --- | --- | --- | --- | --- | --- | --- | --- |
|  | **Pre-implantation** | | **Post-implantation** | | **Pre-implantation** | | **Post-implantation** | |
|  | **SBP** | **DBP** | **SBP** | **DBP** | **SBP** | **DBP** | **SBP** | **DBP** |
|  | (mmHg) | (mmHg) | (mmHg) | (mmHg) | (mmHg) | (mmHg) | (mmHg) | (mmHg) |
| **WKY** | 109 ± 3 | 70 ± 2 | 104 ± 3^*^ | 67 ± 2 | 111 ± 2 | 74 ± 1 | 101 ± 4 | 65 ± 4 |
| **SHR** | 168 ± 2^#^ | 117 ± 1^#^ | 167 ± 4^#^ | 112 ± 2^#^ | 176 ± 4^#^ | 121 ± 4^#^ | 129 ± 10^#*$^ | 85 ± 9^*$^ |
